# Prevalence and resistance mutations of non-B HIV-1 subtypes among immigrants in Southern Spain along the decade 2000-2010

**DOI:** 10.1186/1743-422X-8-416

**Published:** 2011-08-26

**Authors:** Beatriz de Felipe, Pilar Pérez-Romero, María Abad-Fernández, Felipe Fernandez-Cuenca, Francisco J Martinez-Fernandez, Mónica Trastoy, Rosario del Carmen Mata, Luis F López-Cortés, Manuel Leal, Pompeyo Viciana, Alejandro Vallejo

**Affiliations:** 1 Infectious Diseases Service, IBIS, Hospital Univeritario Virgen del Rocio, Seville, Spain; 2 Laboratory of Immunovirology, Infectious Diseases Service, Instituto Ramón y Cajal de Investigaciones Cientificas (IRYCIS), Hospital Universitario Ramón y Cajal, Madrid, Spain; 3 Microbiology Service Hospital Virgen Macarena Seville, Spain; 4 Laboratory of Immunovirology, Infectious Diseases Service, IBIS, Virgen del Rocío University Hospital, Seville, Spain

**Keywords:** non-B HIV-1 subtypes, immigrant, Spain, resistance mutation

## Abstract

**Background:**

Most of the non-B HIV-1 subtypes are predominant in Sub-Saharan Africa and India although they have been found worldwide. In the last decade, immigration from these areas has increased considerably in Spain. The objective of this study was to evaluate the prevalence of non-B subtypes circulating in a cohort of HIV-1-infected immigrants in Seville, Southern Spain and to identify drug resistance-associated mutations.

**Methods:**

Complete protease and first 220 codons of the reverse transcriptase coding regions were amplified and sequenced by population sequencing. HIV-1 subtypes were determined using Stanford University Drug Resistance Database, and phylogenetic analysis was performed comparing multiple reported sequences. Drug resistance mutations were defined according to the International AIDS Society-USA.

**Results:**

From 2000 to 2010 a total of 1,089 newly diagnosed HIV-1-infected patients were enrolled in our cohort. Of these, 121 were immigrants, of which 98 had ethical approval and informed consent to include in our study. Twenty-nine immigrants (29/98, 29.6%) were infected with non-B subtypes, of which 15/29 (51.7%) were CRF02-AG, mostly from Sub-Saharan Africa, and 2/29 (6.9%) were CRF01-AE from Eastern Europe. A, C, F, J and G subtypes from Eastern Europe, Central-South America and Sub-Saharan Africa were also present. Some others harboured recombinant forms CRF02-AG/CRF01-AE, CRF2-AG/G and F/B, B/C, and K/G, in PR and RT-coding regions. Patients infected with non-B subtypes showed a high frequency of minor protease inhibitor resistance mutations, M36I, L63P, and K20R/I. Only one patient, CRF02_AG, showed major resistance mutation L90M. Major RT inhibitor resistance mutations K70R and A98G were present in one patient with subtype G, L100I in one patient with CRF01_AE, and K103N in another patient with CRF01_AE. Three patients had other mutations such as V118I, E138A and V90I.

**Conclusions:**

The circulation of non-B subtypes has significantly increased in Southern Spain during the last decade, with 29.6% prevalence, in association with demographic changes among immigrants. This could be an issue in the treatment and management of these patients. Resistance mutations have been detected in these patients with a prevalence of 7% among treatment-naïve patients compared with the 21% detected among patients under HAART or during treatment interruption.

## Background

Human immunodeficiency virus type 1 (HIV-1) is the major pathogen responsible for the AIDS pandemic. Several genetic variants can be recognized within HIV-1 group M, including nine subtypes (A through K), at least 43 major circulating recombinants forms (CRFs), and multiple unique recombinant forms (URFs) (http://www.hiv.lanl.gov). The prevalence of HIV-1 subtypes varies greatly depending on the geographic region. Subtype B is predominant in North America and Western Europe, including Spain, although is responsible for only 10% of global infections [1]. Non-B HIV-1 subtypes and its recombinants, such as subtype C, A, CRF01_AE or CRF02_AG, are prevalent in Sub-Saharan Africa, Asia and Eastern Europe [2]. These subtypes cause up to 90% of the 36 million estimated infections, playing an important role in the HIV-1 pandemic [3,4]. Human migration produced in the last decade has contributed to the current spread of non-B subtypes in developed countries [5-7].

The extensive variability of HIV-1 has a potential impact on epidemiology, diagnosis, therapy and prevention of infection. In fact, faster progression to AIDS among individuals infected with non-B subtypes or recombinant variants enhances the importance of identifying these strains [8,9]. In addition, differences in the sensitivity to antiretrovirals in patients infected with non-B subtypes due to the high prevalence of polymorphisms in protease (PR) and/or reverse transcriptase (RT) associated with resistance to antiretroviral therapy has to be taken into account [10-16].

Finally, diagnostic tests, including viral load measurements, might be affected by the diversity of HIV-1 strains [17,18]. Therefore, HIV-1 subtype characterization is becoming an important aspect to adequate clinical management of HIV-1-infected individuals [3]. Sub-Saharan population in Spain has increased in recent years. Around 10-15% of HIV-1-infected immigrants are characterized with non-B subtype during the first medical evaluation [19-21].

Thus, the objective of this study was to analyse the prevalence of non-B subtypes in a cohort of HIV-1-infected immigrants in Southern Spain from 2000 to 2010, and to characterize drug resistance mutations associated to PR and RT.

## Methods

### Patients

From January 2000 to December 2010 a total of 1,089 new HIV-1-infected patients were included in the dynamic open cohort of the Infectious Diseases Service at the University Hospital Virgen del Rocio located in Seville, Southern Spain. Of these, 121 (11.1%) were HIV-1 infected immigrants. For the present retrospective study, immigrant patients of any nationality with available samples were selected. The study was approved by the Ethical Committee of the Hospital. A total of 98 patients were included and all signed an informed consent.

### Laboratory determinations

CD4^+ ^T cell count was determined in fresh samples by flow cytometry. Plasma HIV-1 RNA was measured by quantitative PCR (HIV Monitor™ Test Kit, Roche Molecular System, Hoffman-La Roche, Basel, Switzerland), according to the manufacturer's instructions. This assay has a lower detection limit of 50 HIV-1 RNA copies/ml.

### Subtyping and drug resistance profile

For genetic analysis, in patients with detectable viral load, HIV-1 RNA was extracted from plasma using a viral RNA purification kit (Qiagen, Diagnostics, Barcelona, Spain). cDNA was synthesized by avian myeloblastosis virus (AMV) reverse transcriptase. In patients with undetectable viral load and undergoing antiretroviral treatment at the time of the study, HIV-1 proviral DNA from cryopreserved peripheral blood mononuclear cells (PBMCs) isolated from heparinized blood by Ficoll density-gradient centrifugation, were used for DNA extraction (Qiagen DNA blood kit, Diagnostics, Barcelona, Spain), following the manufacturer's instructions.

PCR reactions were carried out in 50 μL final volumes containing 10 mM Tris HCl pH 8.8, 50 mM KCl, 1.5 mM MgCl_2_, 0.1% Triton X-100, 0,2 mM of each dNTP, 0.7 units Taq polymerase (Eurotaq, Euroclone, S.p.A, Syziano, Italy), 0.5 μM of each primer, and 1 μg purified DNA. Cycling parameters were 94°C for 5 min followed by 35 cycles of 94°C for 15 s, 55°C for 15 s and 72°C for 30 s, followed by a 10 min hold at 72°C. Identical conditions were used for the nested PCR. Outer primers PRF2 (5'-cagaagagagtctcaggtttggg-3') and PRF5 (5'-tggagtattgtatggattttcagg-3') for the PR-coding region, and 47RV (5'-gtattagtaggacctacacct-3') and Pol18 (5'-agactcacaatatgca-3') for the RT-coding region, were used. Inner primers Pol10 (5'-ccctcaagggcaggagc-3') and Pol14 (5'-gggccatccattcctgg-3') for the PR-coding region, and A35 (5'-ttggttgcactttaaattttcccattagtcctatt-3') and Lp2 (5'-atcaggatgagattcataacccatcca-3') for RT-coding region, were used. A fragment of 450 bp encompassing the entire PR-coding region and a fragment of 670 bp that includes the first 220 codons of the RT-coding region were generated by nested PCR.

Sequencing of PCR purified amplicons was performed using Applied Biosystems 310 Sequencer and BigDye deoxy terminator procedure as specified by the manufacturer.

Subtyping of the PR and RT sequences was determined using the Stanford University Drug Resistance Database (http://hivdb.stanford.edu). Afterwards, the PR and RT sequences were aligned together with other HIV-1 group M reported sequences available in the GenBank by computer software CLUSTAL W. Phylogenetic trees were generated using the Neighbour-Joining method and bootstrap re-sampling of multiple alignments (1,000 data sets), included in the CLC DNA Workbench software, was employed to test the tree robustness. Drug resistance profiles in PR and RT-coding regions were defined according to the International AIDS Society-USA.

### Statistical Analysis

Continuous variables are shown as median [interquartile range (IQR)], and categorical variables as number of cases (percentage). Chi square test were used to analyze differences between categorical variables. The statistical analysis was performed using the Statistical Package for the Social Sciences software (SPSS 16.0, Chicago, Illinois, USA).

### Sequence Accession Number

GenBank accession numbers for consensus sequences: AM000054 (A); K03455 (B); AY563172 (C); DQ189088 (F); AY017457 (G); EF614151 (J); AJ249239 (K); FM252023 (CRF01_AE); FN557324 (CRF02_AG). Protease sequences: JF338626-JF338629 (PR_SE10-PR_SE13), JF338630-JF338633 (PR_SE15-PR_SE18), JF338634-JF338641 (PR_SE20-PR_SE27), JF338642-JF338653 (PR_SE29-PR_SE40), JF338653 (PR_SE40), JF338654-JF338658 (PR_SE42-PR_SE46), JF338659-JF338663 (PR_SE48-PR_SE52), JF338664-JF338686 (PR_SE54-PR_SE76), JF338687-JF338690 (PR_SE78-PR_SE81), JF338691-JF338716 (PR_SE90-PR_SE115). RT sequences: JF338717-JF338719 (RT_SE10-RT_SE12), JF338720-JF338731 (RT_SE14-RT_SE25), JF338732-JF338744 (RT_SE27-RT_SE39), JF338745-JF338761 (RT_SE41-RT_SE57), JF338762-JF338780 (RT_SE59-RT_SE77), JF338781 (RT_SE79), JF338782-JF338807 (RT_SE90-RT_SE115).

## Results and discussion

### Patients' characteristics

Baseline characteristics of the 98 immigrant individuals studied are summarized in Table 1. The study population was grouped into seven categories according to their home countries: Central-South America (Argentina, Peru, Brazil, Colombia, Cuba, Bolivia, Ecuador, El Salvador, Guatemala, Panama, Paraguay, Dominican Republic and Venezuela), Western Europe (France, Italy, Portugal, Belgium, Germany and Ireland), Sub-Saharan Africa (Nigeria, Cameroon, Angola, Uganda, Sierra Leone, Guinea, Cote d'Ivoire), Northern Africa (Morocco), Eastern Europe (Romania, Ukraine and Russia), North America (United States), and Asia (India). The largest immigrant group in Southern Spain was from Central-South America (47/98, 47.9%). In most cases, these individuals were infected with HIV-1 before moving to Spain.

**Table 1:** Characteristics of the 98 HIV-1-infected immigrant individuals studied

Male gender (%)	68 (69.4)
Age (years)	35 [30-38]
CD4^+ ^count (cell/mm^3^)	321 [212-491]
HIV-1 viral load (log_10_IU/mL)	4.28 [1.6-4.8]
Naïve ART patients (%)	62 (63,2)
Time in treatment (months)	25.1 [14.2-48.1]
Risk factor (%)	
Heterosexual	41 (41.8)
Bisexual	42 (42,8)
Injecting drug users	7 (7.1)
Others	8 (8.1)
Geographical Region (%)	
Central-South America	47 (47.9)
Western Europe	14 (14,3)
Sub-Saharan Africa	19 (19.4)
Northern Africa	5 (5.1)
Eastern Europe	10 (10.2)
North America	1 (1)
Asia	1 (1)
Not determined	1 (1)

**Figure 1:**
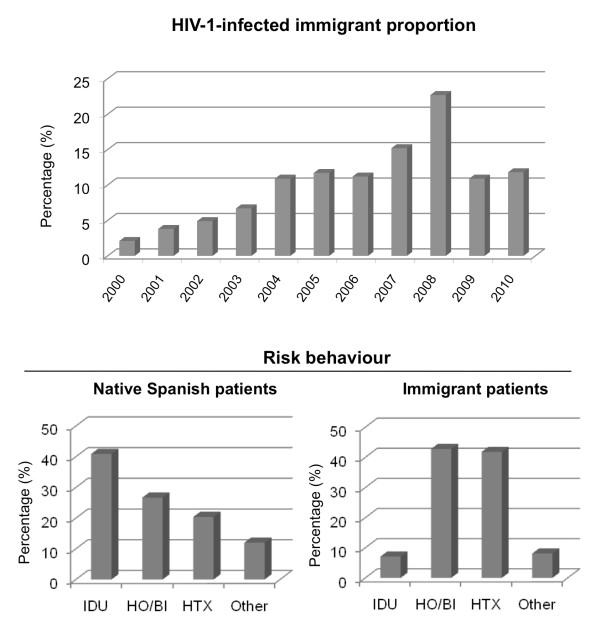
**Proportion of HIV-1-infected immigrants from 2000 to 2010 in Southern Spain, and comparison of risk behavior between native Spanish and immigrant patients**. IDU, injecting drug users; HO/BI, homosexual/bisexual; HTX, heterosexual.

The proportion of immigrants in our cohort of HIV-1-infected patients from 2000 to 2010 is shown in Figure 1. The proportion has been rising year by year from 2.1% in 2000 to around 12% in 2010. Notably, there has been a marked progressive increase up to 2008, which exceeded 20% (Figure 1). Regarding risk behaviour, injecting drug use was the most common route of transmission among native Spanish patients (around 40% vs. 7% among immigrants, p < 0.001 Chi Square test), while sexual transmission (hetero plus homosexual) was predominant (80% vs. 42% among native Spanish patients, p < 0.001 Chi Square test) among immigrants (Figure 1).

### Subtype analysis

Both PR and RT sequences were obtained for 87 patients, while only PR or RT sequences were obtained for seven and four individuals, respectively. Twenty-nine of the overall sequences analyzed belonged to non-B subtypes (29.6%, Table 2). These included A, C, F, G, J, and K subtypes, and recombinant forms, such as CRF02_AG, as well as CRF01_AE. Besides, seven patients harbored different subtypes for each gene, including recombinants B/C, F/B, K/G, CRF02_AG/CRF01_AE, and CRF02_AG/G in PR and RT respectively. According to the geographical origin, the non-B subtype was detected in a total of 79% (15/19) of Sub-Saharan Africans, followed by 60% Eastern Europeans (6/10), 20% Northern Africans (1/5), 10.6% Central-South Americans (5/47) and 7.1% Western Europeans (1/14).

**Table 2:** Characteristics of the immigrant patients infected with HIV-1 non-B subtypes (N = 29)

Patient code	Origin	Treatment	Subtype in PR gene	Protease inhibitor resistance mutations	Subtype in RT gene	RT inhibitor resistance mutations
SE58	EE	HAART	A	M36I, V77I	N.D.	N.D.
SE115	CSA	Naive	B	M36I	C	None
SE71	CSA	HAART	C	M36I	C	V118I
SE73	EE	Naive	F	L10V, K20R, M36I	F	None
SE43	CSA	HAART	F	M36I	B	None
SE99	EE	Naive	F	K20R, M36I	F	None
SE108	CSA	Naive	F	K20R, M36I	F	None
SE37	SSA	Naive	G	K20I, M36I	G	***K70R, A98G***
SE103	SSA	Naive	G	K20I, M36I	G	None
SE31	SSA	Naive	J	K20I, M36I, V77I	J	None
SE91	SSA	Naive	K	M36I, L63P	G	E138A
SE11	SSA	HAART	CRF02_AG	K20I, M36I, L63P	G	None
SE16	SSA	TI	CRF02_AG	K20I, M36I	G	None
SE113	N.D.	Naive	CRF02_AG	K20I, M36I	G	None
SE66	WE	HAART	CRF02_AG	K20I, M36I	CRF02_AG	None
SE59	NAF	HAART	CRF02_AG	K20I, M36I, ***L90M***	CRF02_AG	None
SE67	SSA	HAART	CRF02_AG	K20I, M36I, L63P	CRF02_AG	None
SE49	SSA	Naive	CRF02_AG	K20I, M36I, L63P	CRF02_AG	None
SE50	SSA	TI	CRF02_AG	K20I, M36I, L63P, V77I	CRF02_AG	None
SE33	SSA	TI	CRF02_AG	L10V, K20I, M36I	CRF02_AG	None
SE36	CSA	HAART	CRF02_AG	K20I, M36I	CRF02_AG	None
SE101	SSA	Naive	CRF02_AG	K20I, M36I	CRF02_AG	None
SE110	EE	Naive	CRF02_AG	K20I, M36I	CRF02_AG	None
SE111	SSA	HAART	CRF02_AG	K20I, M36I	CRF02_AG	90I
SE34	SSA	TI	CRF02_AG	K20I, M36I	CRF01_AE	***L100I***
SE96	SSA	Naive	CRF02_AG	K20I, M36I	CRF02_AG	None
SE62	EE	Naive	CRF01_AE	M36I	CRF01_AE	None
SE60	EE	HAART	CRF01_AE	M36I	CRF01_AE	***K103N***
SE19	SSA	HAART	N.D	N.D.	CRF02_AG	None

Protease and RT inhibitor resistance mutations.

CSA, Central-South America; SSA, Sub-Sahara Africa; EE, Eastern Europe; WE, Western Europe; NAF, Northern Africa. HAART, Highly Active Antiretroviral Therapy. TI, treatment interruption. Bold, major resistance. N.D., not determined.

### Phylogenetic analysis

**Figure 2:**
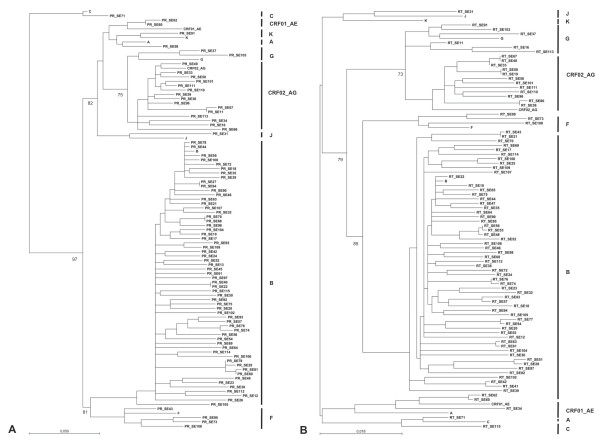
**Phylogenetic analyses derived from protease (A) and reverse transcriptase (B) sequences from HIV-1-infected immigrants from Southern Spain**. Trees were constructed using the neighbor-joining method. Consensus sequences of subtypes A, B, C, F, G, J, K, and circulating recombinant forms CRF02_AG and CRF02_AE are included. Bootstrap values at nodes are shown as percentage of 1000 resamplings (only values greater than 70% are shown). Central-South America (CSA); Sub-Saharan Africa (SSA); Western Europe (WE); Northern Africa (NAF); Eastern Europe (EE); North America (NAM); not determined (ND).

Phylogenetic trees were generated based on PR and RT sequences and included reference sequences representing different HIV-1 subtypes. All sequences clustered within the correspondent subtype, which was concordant with the data provided by Stanford University (Figure 2). Only three of the RT sequences: SE16, SE113, and SE11 corresponding to subtype G were located slightly apart from this subtype although within the same branch. Noteworthy, no sub-clusters were formed based on the geographical origin of the patients. Sequences PR_SE15, PR_SE25, PR_SE51, PR_SE55, RT_SE14, RT_SE15, RT_SE27, RT_SE29, RT_SE45, RT_SE79, were excluded from the phylogenetic analysis due to the limited length that could have influenced the analysis.

### Prevalence of resistance mutations

All patients infected with non-B HIV-1 showed at least one polymorphic mutation in PR (Table 2). Most of these mutations have been frequently found and reported amongst non-B HIV-1 subtypes [14,16]. The M36I mutation in protease was observed in all patients, whilst K20I/R, L63P, V77I and L10V/I mutations were present in 75%, 17.8%, 10.7% and 7.1% of patients, respectively. However, only patient SE59 had the major mutation L90M (1/28; 3.5%). This patient was infected with the recombinant form CRF02_AG and had been under HAART for at least two years.

On the contrary, only six patients showed RT mutations. Three of them (SE37, SE34 and SE60) showed major RT mutations (3/28, 10.7%, table 2). Patient SE37, naïve for antiretroviral treatment, had A98G and K70R major mutations associated with resistance to non-nucleoside RT inhibitors (NNRTI), and nucleoside RT inhibitors (NRTI), respectively. Patient SE34, in treatment interruption, had L100I mutation associated with NNRTI resistance, and finally patient SE60, receiving HAART, had the NNRTI resistance mutation K103N. Minor mutations or polymorphisms in RT were observed in patients SE71, SE91 and SE111 i.e.: V118I, E138A, and V90I mutations respectively.

## Discussion

This study evaluates the prevalence of HIV-1 subtypes and PR and RT drug resistance mutations among HIV-1-infected immigrants living in Southern Spain. Immigration had been gradually increasing since 2000, with a peak of 22% in 2008. Since then, it has been slowly decreasing to 11.8% in 2010. The largest immigrant group was form Central-South America (47.9%) most of them infected with the HIV-1 B subtype. However, up to 25% of immigrants were from countries where HIV-1 non-B subtypes are predominant, as Sub-Saharan Africa with 79% of HIV-1 non-B subtypes (subtypes A, G, J or CRF02_AG), or Eastern Europe with 60% (subtypes A, F, CRF02_AG or CRF01_AE). This marked increase in individuals infected with non-B HIV-1 subtype in our society may have a direct impact on the spread of these subtypes among Spanish native individuals in addition to the changes in appropriate treatment regimes due to differences in genetic sequences amongst the different HIV-1 strains. These results are similar to those found in studies conducted in other regions in Spain, Madrid and Canary Islands [19,20,22].

Previous studies reported a high prevalence of minor resistance mutations in naïve patients infected with HIV-1 non-B subtype that could facilitate the emergence of major mutations [16,23,24]. However, the clinical relevance of minor mutations is unclear. Our results indicated that patients infected with HIV-1 non-B subtypes showed a high frequency of minor PR mutations (polymorphisms) as M36I, L63P, and K20R/I, in contrast to the low proportion of major resistance mutations. In fact, only one patient, subtype CRF02_AG, had L90M mutation probably as a result of receiving antiretroviral therapy during two years (1/28, 3.5%). No difference between the number of patients with major or minor RT mutations (3/28, 10.7% in both cases) was found. Nevertheless, the proportion of patients with major RT mutations was higher compared to patients with major PR mutations.

It is of note that one treatment naïve patient, with G subtype, showed major RT mutations associated with resistance to NRTI (K70R) and NNRTI (A98G). This patient might have acquired these resistance mutations at the moment of infection. One limitation of our study was the impossibility to test plasma samples of antiretroviral-experienced patients before the initiation of antiretroviral therapy to determine primary resistance mutations.

An increasing effort has been done to characterize the difference in antiretroviral therapy responses within HIV-1 non-B subtypes. Although clinical evidence remains limited, response to antiretroviral therapy does not appear to differ significantly among subtypes [3]. In our study, 29 patients showed HIV-1 non-B subtype in PR and/or RT. Eleven of them were under antiretroviral therapy and had good virological and immunological responses. Nevertheless, larger cohorts are necessary to confirm our results. On the other hand, 14 immigrants infected by HIV-1 non-B subtype were naïve for antiretroviral therapy. Most of them with viral load around 4 log HIV-1 copies/ml, and two patients had undetectable viral load for a long period of time (one of them at least for one year). As previous studies have described the failure of laboratory techniques to detect HIV-1 non-B subtypes [25-27], however it would be interesting to establish whether the failure of the diagnosis may be due to a phenotype characteristic of viral load controller in this population.

## Conclusions

The circulation of HIV-1 non-B subtypes among immigrants, with a prevalence of 29.6%, has significantly increased in Southern Spain in the last decade, in association with demographic changes. Spread of CRF02-AG, G and F subtypes, and also some recombinant forms appear to predominate, which is consistent with previous findings [19,20,22,28]. This fact may affect the treatment and management of these patients, and the spread of these subtypes among native Spanish population. Furthermore, a low percentage of the immigrants with the non-B subtypes had major resistance mutations. Prevalence of these resistance mutations varies from 7% among treatment-naïve patients to 21% among patients under HAART or during treatment interruption.

## Competing interests

The authors declare that they have no competing interests.

## Authors' contributions

BF made most of the amplifications, sequencing, drug resistance profiles, sequence alignments and wrote a first draft of the manuscript. PPR, FJMF and FFC made some amplifications and sequencing. MAF helped in alignments of the sequences and revising the manuscript. MT and RCM helped in the management of the patients and collecting samples. LLC, ML, and PV recruited all the patients. AV designed and coordinated the study, helped in the alignment of the sequences and finalized the manuscript in its final form. All authors read and approved the final manuscript.
